# Urban living and chronic diseases in the presence of economic growth: Evidence from a long-term study in southeastern China

**DOI:** 10.3389/fpubh.2022.1042413

**Published:** 2022-12-19

**Authors:** Yixuan Luo, Sailan Wang

**Affiliations:** ^1^Department of Economics and Finance, School of Economics and Management, Tongji University, Shanghai, China; ^2^Department of Physical Examination, Wuyishan Municipal Hospital, Wuyishan, China

**Keywords:** urban development, urban-rural disparity, psychological distress, nutrition shift, type 2 diabetes, public health management

## Abstract

High-speed urban development has brought about an increase in per capita income in low- and middle-income countries (LMICs) as well as the high prevalence rate of chronic diseases. Based on a study of chronic diseases from 2011 to 2021 in southeastern China, we used multivariate adjusted logistic regression method to analyze the effect of urban living on the incidence of typical chronic diseases and the trend of such effect with the improvement of public healthcare system. We adopted potential mediating risk factors of urban lifestyles including body mass index (BMI), frequency of dining out, sedentary time, and psychological distress in the adjusted estimation. Baseline results indicate a positive relationship between living in urban areas and the prevalence of type 2 diabetes, hyperlipidemia, and hypertension. Regarding the mediating factors, psychological distress had the highest positive coefficient (Cr) on type 2 diabetes, hyperlipidemia, and hypertension (Cr: 0.4881–0.7084), followed by BMI (Cr: 0.1042–0.1617) and frequency of dining out (Cr: 0.0311–0.0478), and finally, sedentary time (Cr: 0.0103–0.0147). However, regression results on the follow-up survey reveal that trend in the impact of living in urban areas on chronic disease diminished as the level of the healthcare system improved. Additionally, urban living was more positively correlated with the incidence of metabolic disease than with the incidence of cardiovascular disease and cancer. Our findings provide empirical evidence that future urban health planning in LMICs should pay sustained attention to upgrading the level of public health infrastructure covering urban residents as well as rural-to-urban migrants, constructing a long-term dynamic system of chronic disease prevention and control, and regularly monitoring the mental health problems of residents in order to interrupt the process of urban chronic disease prevalence in an early stage.

## Introduction

Urban development is a key indicator of global economic development. A number of countries and regions, particularly low- and middle-income countries (LMICs), have witnessed a significant upward trend in urban development in recent decades. The initial urban development process is driven by industrialization, in which enterprises that are engaged in production activities tend to concentrate the development geographically in order to acquire the economic benefits of agglomeration. High-speed urban development creates development opportunities for these countries and brings a series of changes to people's diet structures, lifestyles, and living environments. Simultaneously, the high prevalence of chronic diseases in such regions with remarkable economic growth has attracted academic attention (1).

Due to socioeconomic, cultural, and political differences, the impact of residence location on human health is complex and non-homogeneous in both temporal and regional dimensions (2). Although the relationship between urban lifestyles and chronic diseases in LMICs is inconclusive (3), there remain different views on this controversial issue. Previous research highlighted that urban living was accompanied by improvements in micronutrient intake and status, but also by increases in several risk factors for non-communicable diseases (4). According to an analysis of nationally representative data on LMICs, women living in higher per capita income and urban areas are associated with a higher probability of being overweight (5). Conversely, there is also the view that the income gradient in overweight individuals will go through a positive to negative change in the process of economic growth (6). Urban natural environments can even be a nature-based solution for improving public health (7).

As China's economy began to grow exponentially since 1978 when the “Reform and Opening-up” policy was launched, millions of rural people continued to migrate to cities (8), and between 2011 and 2021, the proportion of China's population living in urban areas increased from 51.83% to 63.89% (9). China's planning for cities began in the 1990s, and by the 21^st^ century, cities had grown much larger than was expected at the former stage of city planning, which has led to a situation in which infrastructure and supporting policies have deviated from the actual population capacity and scale of cities (10). Nonetheless, the Chinese government is continuously committed to upgrading the basic health system, and the healthcare system reform undertaken by the Chinese government since 2009 has made commendable achievements, including the expansion of social health insurance, the reform of public hospitals, strengthening of primary health care, and the establishment of multiple initiatives to relax constraints on the growth of the private health care sector (11).

In this study, we explored the comprehensive mechanism of urban living on the prevalence of chronic diseases over a decade of rapid economic development in China (2011–2021). The results show that living in urban areas has significant positive effects on the incidence of typical chronic diseases, including type 2 diabetes, hyperlipidemia, and hypertension, but this effect tends to diminish as the level of urban basic medical standards improves. This finding underlines the significance of building parallel public health prevention and treatment systems in cities, and provides empirical evidence toward the critical role of welfare systems covering urban residents as well as rural-to-urban migrants. According to the projection of global urbanization (12), China's urbanization trend may continue for another four to five decades. Likewise, challenges may be posed in other LMICs with demographic structure similar to China. In this regard, research on the integrated pathogenic mechanism of urban living on chronic diseases should be multifaceted, worldwide, and simultaneously, an urgent matter.

This study makes several contributions to the literature. First, the relationship between living in urban areas and chronic disease has been studied by many authors, but most have focused on a specific mediator (e.g., nutritional transition, depression) (13–18). We adopted a broader range of potential mediators from the literature and make the contributions between risk factors comparable. Second, some previous studies were based on a set of cross-sectional data (19–21). This approach can observe the effect at a particular period, while it may ignore capturing the long-term trends; hence, we provided a longitudinal analysis using data from different time points and classified the impact of urban living on different types of chronic diseases, in an effort to develop a more targeted prevention and control system for chronic diseases. Finally, we discuss how non-conventional risk factors (mental illness) change the understanding of epidemiology in the context of urban development and what potential technological and demographic pressures they may pose on the capacity and efficiency of the future urban healthcare system (3). We aim to use this empirical study to provide systematic analysis and scientific insights toward modern cities' chronic disease prevention in developing countries with rapid economic growth as well as sustained population increase.

## Potential risk factors for chronic diseases posed by urban living

We divided risk factors brought about by urban living into two types from the literature: physical and mental aspects. Physical factors refer to nutritional transitions and dietary habits differences caused by growth in per capita income. The acceleration of nutritional transition in cities contributing to their rapid increase in chronic diseases is based on the penetration of modern food systems into society, where the main differences in diet composition have been the increase in refined carbohydrates, the addition of sweeteners, edible oils, and foods of animal origin, and the reduction of pulses and other vegetables. This shift has led to a sharp increase in body mass index (BMI) and waist circumference, which has caused the prevalence of chronic diseases including diabetes and hypertension (22). There may also be a correlation between the increased frequency of dining out due to urban development and the risk of diabetes and hyperglycemia. Increased frequency of dining out is likely to lead to higher fat, cholesterol, and carbohydrate intake, and lower micronutrient intake, particularly vitamin C, calcium, and iron (16).

Another physical risk is sedentariness and physical inactivity owing to technological advances. The prosperity of modern technology provides convenient transportation and makes sedentary habits common among urban residents (23). In a cross-sectional study, for older adults (age ≥ 60 years), the probability of having ≥2 chronic diseases was 76% higher for men who were sedentary for >4 h/day, and 82% higher for women who were sedentary for >4 h/day (24). Moreover, children with a chronic disease were found to be sedentary for an average of 10.2 ± 1.4 h/day, accounting for 76.5 ± 7.1% of the average time activity per day (25).

In addition to physical factors, mental problem is another pivotal mediating risk factor posed by urban living. Areas with high population densities are characterized by higher air pollution and noise, leading to a higher incidence rate of psychiatric disorders (26). Furthermore, the underlying social determinants of mental health, such as housing instability and food insecurity, unemployment, welfare and healthcare disparities, and educational inequity that are incompatible with the size of urban populations are becoming increasingly diversified and complex within the context of urban development (27–29). Prevalence rates of psychological distress, anxiety disorders, and post-traumatic stress disorder (PTSD) are higher in urban settings, where urban living influences the biological stress regulation pathways (17, 26). These mental problems brought about by urban environmental and socialization factors are potential mediators of urban living on the prevalence of typical chronic diseases. The accumulation of chronic stress may directly or indirectly result in the development of various diseases such as atherosclerosis, fatty liver disease (including non-alcoholic), and inflammation (30).

Although urban development has caused an increasing prevalence of chronic diseases in LMICs, it is undeniable that modernization has also accelerated the evolution of the healthcare system in the presence of economic growth. An indication of this is the four cross-cutting strategies proposed by the US Centers for Disease Control and Prevention (CDC): (1) epidemiology and surveillance to monitor trends to inform programs; (2) environmental approaches to promote health and support healthy behaviors; (3) health system interventions to improve effective utilization of clinical and other preventive services; and (4) community resources linked to clinical services to sustain chronic disease management. Combining these four strategies could prevent the onset of chronic diseases and promote early detection and mitigation of disease (31). While China is making progress in strengthening its primary healthcare system, there is more room for improvement in terms of controlling risk factors such as hypertension and diabetes. As China's healthcare reforms deepen, there is an opportunity to build an integrated, collaborative primary healthcare system that will better serve one-fifth of the world's population (32).

## Materials and methods

### Data

Data used in this study were obtained from two cross-sectional surveys. The baseline survey was conducted in Wuyishan City, Fujian Province, southeast China, from June 2011 to January 2012. A total of 7,268 residents in 54 communities of the city aged 40 to 75 years old were selected using a random sampling method, among them 6,589 residents agreed to participate and signed the informed consent form. Participants were interviewed using questionnaires on basic information concerning age, sex, marital status, employment status, smoking habits, emotional conditions, dietary habits, sedentary time, past medical history, and underwent a physical examination in Wuyishan Municipal Hospital, including BMI, waist circumference, fasting and 2-h postprandial plasma glucose levels, blood pressure (systolic and diastolic), lipid levels (total cholesterol, low-density lipoprotein cholesterol, high-density lipoprotein cholesterol, and triglyceride). We excluded 181 participants in basic estimation and 645 participants in multivariate adjusted estimation due to incomplete information.

A follow-up visit was supplemented in 2021 for the 6,589 participants in the baseline survey, whose information was recollected in combination with telephone callbacks and a citywide medical network system database. Among them, 328 deceased and 178 with missing key information were excluded. We used government-defined urban and rural communities to classify the residence location of the participants, where the urban group includes people living in and around the centers of government-defined urban communities (33). First, compared to major metropolises in China, our study site was a typical small to medium-scale city. The urban development progress of the city has been in its primary stage and there is no significant aggregation effect of poor populations observed around urban communities. Second, adopting the classification standard consistent with government-defined urban and rural communities allows for identifying the type of healthcare services provided to the participants. Key explanatory variables (excluding discontinuous variables) in both datasets were winsorized at the 1% and 99% quartiles to prevent disturbances from extreme values.

### Conceptual model

We used the multivariate adjusted logistic regression method to explore the comprehensive mechanism of urban living on the incidence of typical chronic diseases, which essentially refers to the study on the association between urban living and non-communicable diseases (5, 34), and expanded on this in conjunction with analysis of aforementioned factors:


(1) $$\begin{array}{l} \text{P}({\text{Y}}_{\text{ij}}=1)=\frac{\text{exp}(\text{Z})}{1+\text{exp}(\text{Z})},\\ \;\text{Z}=\text{α}\;+\;{\text{β}}_{1}\text{U}{\text{L}}_{\text{i}}\;+{\text{A}}_{\text{i}}^{\prime }\;{\text{β}}_{2}+{\text{Z}}_{\text{i}}^{\prime }\;\text{γ}\;+\;{\text{ε}}_{\text{i}} \end{array}$$

where **Y**_**ij**_ represents whether participant **i** has **j** chronic diseases (yes = 1, 0 otherwise), **UL**_**i**_ represents residence location of participant (living in urban areas = 1, 0 otherwise). Vector $A_{i}^{\prime }$ is a set of mediating variables, whose risk level differences among participants were potentially posed by urban-rural living disparity, including BMI, frequency of dining out, sedentary time, and psychological distress. $Z_{i}^{\prime }$ is control variable vector including gender, age, marital status, and smoking habits (19, 35).

When analyzing the relationship between urban living and chronic diseases, BMI, frequency of dining out, sedentary time, and psychological distress in the $A_{i}^{\prime }$ vector were excluded due to potential mediation (36). In further examination of the specific effects of mediating risk factors, residence location can be adopted to capture unobserved fixed effects including level of medical services received by participants and degree of exposure to environmental changes. Variables classified as unmodifiable risk factors in the control variables vector were included in both basic and multivariate adjusted estimations (37).

## Results

### Descriptive statistics

In the multivariate adjusted regression based on the 2011 baseline survey, we eliminated 645 outliers due to missing values of key explanatory variables (incomplete physical examination or omission of questionnaire items) from 6,589 participants, and a total of 5,944 were eventually included in both basic and multivariate adjusted estimations. Characteristics of participants are shown in Table 1. In the baseline survey, participants were aged 40 to 75 years, with a mean value of 52.9717 years (St. Dev. = 8.8507); the proportion of the total sample from the urban area was 52.78%, which is in accordance with the local population distribution pattern.

**Table 1:** Participant characteristics in the 2011 baseline survey.

** Variables **	** Full sample **		** Urban **		** Rural **	
	** Mean **	**St. Dev**.	** Mean **	**St. Dev**.	** Mean **	**St. Dev**.
Age (years)	52.9717	8.8507	53.3079	9.0659	52.5960	8.5898
Sex (Men = 1, Women = 2)	1.5384	0.4986	1.5952	0.4909	1.4749	0.4995
Residence location (Urban areas = 1, 0 Otherwise)	0.5278	0.4993	-	-	-	-
Marital status (Married = 1, Divorced or living alone = 0)	0.8728	0.3332	0.8690	0.3375	0.8771	0.3284
Employment status (Employed = 1, 0 Otherwise)	0.4539	0.4996	0.4734	0.5019	0.4321	0.4962
Cigarette smoking (Current smoking = 1, 0 otherwise)	0.2397	0.4935	0.1741	0.4493	0.3131	0.5292
BMI (Weight/Height^2^ [kg/m^2^])	24.2575	3.1979	24.4138	3.1589	24.0829	3.2326
Frequency of dining out (times a week)	1.0288	3.2691	1.3586	3.5335	0.6601	2.9022
Sedentary time (hours a week)	24.6476	14.9353	25.6552	17.7788	23.5215	10.8063
Psychological distress level (Range: 1–4^*^)	1.2086	0.2339	1.2176	0.2577	1.1984	0.2035
Type 2 diabetes^a^ (Yes = 1, 0 Otherwise)	0.0624	0.2419	0.0842	0.2777	0.0381	0.1915
Hyperlipidemia^b^ (Yes = 1, 0 Otherwise)	0.0611	0.2395	0.0873	0.2824	0.0317	0.1752
Hypertension^c^ (Yes = 1, 0 Otherwise)	0.0597	0.2370	0.0803	0.2718	0.0367	0.1880
Observations	5,944		3,137		2,807	

* Psychological distress level questionnaire referred to relevant studies (26, 62). We took the average value of the nine related questions (Supplementary material).

a Defined as fasting plasma glucose 7.0 mmol/L or 2-h postprandial plasma glucose 11.1 mmol/L referring to World Health Organization (WHO).

b Defined by the US National Cholesterol Education Program (NCEP).

c Defined as mean systolic blood pressure (SBP) 140 mmHg and/or mean diastolic blood pressure (DBP) 90 mmHg referring to WHO.

The prevalence rates of type 2 diabetes, hyperlipidemia, and hypertension were 6.24%, 6.11%, and 5.97% in the full sample. Specifically, the prevalence rates were 8.42%, 8.73%, and 8.03%, for the urban population and 3.81%, 3.17%, and 3.67%, for the rural population. Regarding the mediating factors, the urban population had 1.37%, 1.06%, 9.07%, and 1.60% higher BMI, frequency of dining out, sedentary time, and psychological distress, respectively, than the rural population. Results of descriptive statistics have initially revealed a potential relationship between living in urban areas and chronic diseases. We have also provided baseline characteristics of the group that completed the follow-up visit and the group that lost to the follow-up visit due to decease (Supplementary material). Compared to the follow-up group, the deceased group had 18.98%, 131%, and 93.87% higher age, the prevalence rates of type 2 diabetes and hypertension, whilst 44.76% and 8.40% lower rates of marriage and employment, respectively. These results imply that aging, underlying chronic diseases, and the status of living alone impose substantial burdens on older people in China.

### Baseline results

Tables 2, 3 present the baseline results. Coefficients (Cr) of residence location for type 2 diabetes (Cr = 0.8148, *p* < 0.01), hyperlipidemia (Cr = 1.1550, *p* < 0.01), and hypertension (Cr = 0.7498, *p* < 0.01) indicate a significantly positive relationship between urban living and the prevalence of chronic diseases (Table 2). For the control variables, age was positively correlated with the dependent variables (Cr: 0.0085–0.0660), which is consistent with previous studies (15, 18) and implies the challenges to the efficiency of the healthcare system posed by China's remarkable demographic shift (38).

**Table 2:** The association of urban living with chronic diseases.

** Variables **	** VIF† **	** Type 2 diabetes **	** Hyperlipidemia **	** Hypertension **
		** Coefficient (standard error) **	** Coefficient (standard error) **	** Coefficient (standard error) **
Residence location	1.03	0.8148^***^ (0.1162)	1.1550^***^ (0.1264)	0.7498^***^ (0.1240)
Age	1.05	0.0177^***^ (0.0058)	0.0085^*^ (0.0044)	0.0660^***^ (0.0063)
Sex	1.42	−0.0244 (0.1228)	−0.1863 (0.1352)	0.1634 (0.1311)
Marital status	1.03	0.0307 (0.1484)	−0.1065 (0.1510)	0.1697 (0.1583)
Employment status	1.11	−0.5067^***^ (0.1202)	−0.0188 (0.1095)	−0.0667 (0.1317)
Cigarette smoking	1.36	−0.0807 (0.1372)	0.0279 (0.1811)	−0.0310 (0.1362)
Observations		6408	6408	6408
Prob > chi^2^		0.0000	0.0000	0.0000

Robust standard errors in parentheses;

*** *p* < 0.01,

** *p* < 0.05,

* *p* < 0.1.

† Variance inflation factors (VIF) were examined for each variable to exclude the problem of multicollinearity (range: 1–1.5).

**Table 3:** Potential mediators of urban living on chronic diseases.

** Variables **	** Type 2 diabetes **	** Hyperlipidemia **	** Hypertension **
	** Coefficient (standard error) **	** Coefficient (standard error) **	** Coefficient (standard error) **
BMI	0.1042^***^ (0.0163)	0.1617^***^ (0.0162)	0.1076^***^ (0.0169)
Frequency of dining out	0.0420^**^ (0.0175)	0.0311^**^ (0.0148)	0.0478^***^ (0.0175)
Sedentary time	0.0103^*^ (0.0054)	0.0147^***^ (0.0053)	0.0090 (0.0056)
Psychological distress level	0.4881^**^ (0.1965)	0.7084^***^ (0.1735)	0.5154^***^ (0.1975)
Residence location	0.6852^***^ (0.1235)	0.9631^***^ (0.1295)	0.6684^***^ (0.1263)
Age	0.0699^***^ (0.0064)	0.0408^***^ (0.0065)	0.0693^***^ (0.0065)
Sex	0.1793 (0.1288)	−0.0349 (0.1360)	0.1898 (0.1322)
Marital status	0.3034^*^ (0.1615)	0.0723 (0.1635)	0.2218 (0.1614)
Employment status	−0.0979 (0.1355)	0.2135 (0.1339)	−0.1283 (0.1385)
Cigarette smoking	−0.0223 (0.1312)	0.0867 (0.1438)	−0.0108 (0.1325)
Observations	5,944	5,944	5,944
Prob > chi^2^	0.0000	0.0000	0.0000

Robust standard errors in parentheses;

*** *p* < 0.01,

** *p* < 0.05,

* *p* < 0.1.

In mediating factors, BMI, frequency of dining out, and psychological distress had substantial effects on type 2 diabetes, hyperlipidemia, and hypertension in the multivariable adjusted regression (Table 3). Sedentary time had a significant positive effect on type 2 diabetes and hyperlipidemia, but not on hypertension. Among these risk factors, psychological distress had the highest coefficient for chronic diseases (Cr: 0.4881–0.7084), followed by BMI (Cr: 0.1042–0.1617) and frequency of dining out (Cr: 0.0311–0.0478), and finally, sedentary time (Cr: 0.0103–0.0147). An additional level of psychological stress led to 48.81%, 70.84%, and 51.54% increases in the probability of having type 2 diabetes, hyperlipidemia, and hypertension, respectively.

From the aforementioned analysis, the nutritional shift may be reflected in an increase in residents' BMI or waist circumference (22). To test the robustness of the estimation, participants' waist circumference (cm) was used to replace the core explanatory variable BMI, and the results are reported in Table 4. Compared with the baseline regression, the influence direction and significance of other key explanatory variables did not change considerably, and waist circumference, as a proxy for BMI, also had a significant positive effect on the prevalence of type 2 diabetes, hyperlipidemia, and hypertension.

**Table 4:** Robustness check supporting baseline results using the method of key variable replacement.

** Variables **	** Type 2 diabetes **	** Hyperlipidemia **	** Hypertension **
	** Coefficient (standard error) **	** Coefficient (standard error) **	** Coefficient (standard error) **
Waist circumference	0.0495^***^ (0.0065)	0.0713^***^ (0.0065)	0.0521^***^ (0.0067)
Frequency of dining out	0.0373^**^ (0.0177)	0.0254^*^ (0.0148)	0.0428^**^ (0.0178)
Sedentary time	0.0103^*^ (0.0054)	0.0145^***^ (0.0053)	0.0090 (0.0056)
Psychological distress level	0.4760^**^ (0.1960)	0.6984^***^ (0.1695)	0.5032^**^ (0.1971)
Residence location	0.6359^***^ (0.1239)	0.8924^***^ (0.1298)	0.6164^***^ (0.1267)
Age	0.0632^***^ (0.0064)	0.0315^***^ (0.0065)	0.0623^***^ (0.0066)
Sex	0.3408^***^ (0.1306)	0.2273^*^ (0.1354)	0.3589^***^ (0.1337)
Marital status	0.3246^**^ (0.1627)	0.0997 (0.1648)	0.2437 (0.1626)
Employment status	−0.0985 (0.1364)	0.2143 (0.1350)	−0.1290 (0.1395)
Cigarette smoking	−0.0227 (0.1263)	0.0762 (0.1344)	−0.0111 (0.1272)
Observations	5,943	5,943	5,943
Prob > chi^2^	0.0000	0.0000	0.0000

Robust standard errors in parentheses;

*** *p* < 0.01,

** *p* < 0.05,

* *p* < 0.1.

### Comparative analysis

To analyze trend in the impact of urban development, we supplemented a follow-up survey in 2021. In this survey, telephone interviews, in combination with local medical databases, were used to extend the range of chronic diseases surveyed to include coronary heart disease, and cancer, which are consequential contributors to the burden in older people in LMICs (39, 40). Referring to classification on chronic diseases from literature, coronary heart disease belongs to cardiovascular diseases broad category (41). Investigation of coronary heart disease can be valid complement in addition to hypertension, which is also subcategory of cardiovascular diseases and have been analyzed in the baseline results. Identically, cancer is another representative broad category of chronic diseases supplemented in our follow-up surveys (41). Results of the follow-up surveys are presented in Table 5.

**Table 5:** Results based on the 2021 follow-up survey.

** Variables **	** Type 2 diabetes **	** Coronary heart disease **	** Cancer **
	** Coefficient (standard error) **	** Coefficient (standard error) **	** Coefficient (standard error) **
Residence location	0.6440^***^ (0.1037)	0.4772^***^ (0.1383)	0.3969^***^ (0.1459)
Age	0.0140^***^ (0.0047)	0.0249^***^ (0.0087)	0.0192^***^ (0.0069)
Sex	−0.0830 (0.1147)	−0.2901^*^ (0.1495)	0.5858^***^ (0.1614)
Marital status	0.1233 (0.1432)	0.1249 (0.1955)	0.2792 (0.2239)
Employment status	−0.4035^***^ (0.1033)	−0.5779^***^ (0.1592)	−0.1909 (0.1620)
Cigarette smoking	−0.2352 (0.1434)	−0.1516 (0.1719)	0.1356 (0.0831)
Observations	6,083	6,083	6,083
Prob > chi^2^	0.0000	0.0000	0.0000

Robust standard errors in parentheses;

*** *p* < 0.01,

** *p* < 0.05,

* *p* < 0.1.

A longitudinal comparison of the regression results for the two cross-sectional datasets revealed a 20.96% decrease in the coefficient of living in urban areas for type 2 diabetes (Cr = 0.6440, *p* < 0.01). One potential reason is that enhancements in healthcare prevention and treatment systems weaken the impact of urban lifestyles on the prevalence of chronic diseases (42, 43). At this stage, China tends to be defined more as one of the upper middle-income countries seeking to move toward higher income status rather than “emerging economies” (44). Compared with 2001–2010, the number of local health facilities, medical staff, and medical beds per 1,000 people increased by 40.38%, 75.15%, and 93.02%, respectively, from 2011 to 2020 (45). Specifically, urban-rural differences in the utilization of preventive health services may amplify this weakening trend; the main factors contributing to this phenomenon are differences in family income, education, and rate of disease awareness (46).

Coronary heart disease and cancer were chosen for the cross-comparison to represent chronic diseases indirectly associated with living in urban areas and with higher heterogeneity, uncertainty, and diversity of pathogenesis (47). The results showed an association between coronary heart disease and urban living (Cr = 0.4772, *p* < 0.01), but the degree was lower than that of type 2 diabetes. Likewise, the effect of living in urban areas on hyperlipidemia or type 2 diabetes (both are metabolic diseases) (41) was higher than that of hypertension (cardiovascular disease) (41) in the baseline regression. This empirical evidence supports the view that effects of urban living on cardiovascular diseases are partially transmitted through metabolic diseases. A sustained increase in the prevalence rate of diabetes or hyperlipidemia in the population may lead to a concomitant increase in the incidence of cardiovascular diseases (48–50). Similarly, a positive correlation between urban living and cancer can be concluded, in accordance with the literature (51). However, cancer had the lowest level of association (Cr = 0.3969, *p* < 0.01).

## Discussion

Our findings in this study again verified the role of the government in preventing negative externality issues in the urban development process, and in building a constantly updated and evolving, targeted, integrated healthcare management (52). Results of this empirical investigation also demonstrate that the high prevalence of chronic diseases due to urban development and aging will continue to put tremendous pressure on the capacity and efficiency of health care services in the coming decades. Accordingly, efforts should be made to improve the incidence of chronic diseases through positive corrections to interrupting the long-term processes that cause chronic diseases at an early stage, including changes in the physical fabric through policy interventions (53), and raising awareness rate and prevention knowledge among the population based on the general increase in the population's education level. A comparative study revealed that associations between health status measures and social determinants of health were largely not supported among rural women, but were for their urban counterparts (54). In this regard, multilevel interventions aimed at eliminating systemic social inequalities regarding access to educational and employment opportunities, welfare coverage, and secure housing would have far-reaching implications in urban development (e.g., homeless assistance programs, income supplements, and refundable tax credits for low-income working individuals) (27).

For the public healthcare sector, more emphasis could be placed on mental health guidance, regular monitoring of the psychological status of residents, and results-oriented integration of monitoring results into the future implementation of healthcare system reform. To promote the development of urban healthcare, equalization in public healthcare is an imperative issue that requires to be recognized. Rural-to-urban migrators with higher needs for health services remain a particular group that should be focused on. The “floating” population is affected by more diversified risk factors of urban living and may not have healthcare coverage equal to urban residents (55). In addition to addressing these health challenges involving disturbing disparities in access to medical services, vaccination coverage, and accident and injury insurance among China's rural-to-urban migrated population, innovation in health policies should also focus on migrants' needs for primary care, preventive health check-ups, and early diagnoses of cancers (20, 56, 57).

The private healthcare sector has become an increasingly important complement to improve the adequacy and equality of China's healthcare system since the healthcare system reform. However, strict regulation and insufficient risk-management capacity have continued to hamper private sector involvement in health services production and insurance. Data from the China Health and Retirement Longitudinal Study have investigated that although patients with health insurance and higher socio-economic status were more likely to be aware of health service provider's ownership, they preferred public providers over private providers (58). According to our findings, while urban development accelerates the emergence of economies, urban dietary habits and lifestyles have simultaneously brought complex superimposed effects among chronic disease risk factors for modern humans. To meet the enormous pressure in demand for healthcare in cities, a compromise model in which competing private providers are non-negligible participants in the healthcare system, but in which the government intervenes in such a way as to attain both a high degree of healthcare equalization and to avoid the “market failure” in an unregulated private system may be the optimal choice (59).

Increasing incomes and urban development are driving a global dietary transition, with traditional diets being replaced by diets high in refined sugars, fats, oils, and meat. Modern urban diets significantly increase the incidence of chronic diseases such as type 2 diabetes, coronary mortality, and cancer in addition to the higher growth of greenhouse gas emissions than Mediterranean, pescatarian, and vegetarian diets. These trends will be a major contributor to an 80% increase in global agricultural greenhouse gas emissions by 2050, and therefore, implementing solutions that address the strong “diet-environment-health” nexus is a global issue (60). The guidance of a rational diet structure in public health management will subsequently benefit the socio-economical goals of food security, sustainable use of land resources, and ecological protection in 21^st^ century urban planning.

Although living in cities is a global trend and is most prevalent in the LMICs, cities of LMICs are exceptionally ill-prepared for the explosion in urban living (61). Our study provides experiences for LMICs undergoing high-speed economic growth or economic transition that the urban primary healthcare system construction should be the focus of urban welfare system planning. In the future, this will become the solid foundation for releasing the pressure on urban healthcare services brought about by the explosion in urban residents. Further, owing to heterogeneous presentations of chronic diseases among LMICs, a better understanding of the epidemiology is urgently needed to include non-conventional risk factors concerning anxiety disorders, sleep insufficiency, and stress as factors mediating urbanization and chronic diseases (3, 62, 63). As our research has shown, psychological stress may be another necessary concern in addition to income body weight gradients in LMICs' rapid urbanization process (5, 6), whose contribution to the incidence of type 2 diabetes, hyperlipidemia, and hypertension has been proved to be 3.68, 3.38, and 3.79 times higher than nutrition transition, respectively.

This study presents some limitations. Although we compared the variability in the relationship between urban living and typical chronic diseases, future research continues to be required on the mechanisms of effects on cardiovascular diseases and cancer. In addition, factors associated with neighborhood effects including assets value, the number of social security beneficiaries, and social cohesion could extend the study on the incidence of chronic diseases in the context of urban living (17, 21). Another potential study limitation is that the follow-up survey supplemented was still based on cross-sectional data with infrastructural constraints. In the next phase, we will consider a dynamic approach to include more longitudinal information on subjects and systematically classify the relationship between living in urban areas and chronic diseases.

## Data availability statement

The datasets presented in this article are not readily available because participants data are used for scientific research only, further requests for data are required to obtain approval from the survey project committee. Requests to access the datasets should be directed to 5134wsl@163.com.

## Ethics statement

The studies involving human participants were reviewed and approved by the Research Protection Committee of Wuyishan Municipal Hospital. All participants signed an informed consent form before information collection. The patients/participants provided their written informed consent to participate in this study.

## Author contributions

YL: writing and editing of the manuscript. SW: data collection and co-editing of the manuscript. Both authors contributed to the article and approved the submitted version.
