# Supplementary material for: Urban living and chronic diseases in the presence of economic growth: Evidence from a long-term study in southeastern China
Source: Front Public Health. 2022 Dec 19;10:1042413. doi: 10.3389/fpubh.2022.1042413 (PMC9806235; doi:10.3389/fpubh.2022.1042413)
Supplement: Supplementary file 1 [file Table_1.DOCX]

Supplementary Material

# Supplementary Tables

Appendix 1. Questionnaire on psychological distress level

| Questions | Level 1 | Level 2 | Level 3 | Level 4 |
| --- | --- | --- | --- | --- |
| Do you feel you have had no  interest in doing anything  and no fun in the last 2 weeks? | No | A few days but not many | More than a week | Almost every day |
| Have you felt down, depressed,  or hopeless in the last 2 weeks? | No | A few days but not many | More than a week | Almost every day |
| Have you been feeling unmotivated  in the last 2 weeks? | No | A few days but not many | More than a week | Almost every day |
| Have you lost your appetite or eaten  too much in the last 2 weeks? | No | A few days but not many | More than a week | Almost every day |
| Have you been dissatisfied with  yourself, or feel like a failure in  the last 2 weeks? | No | A few days but not many | More than a week | Almost every day |
| Do you feel that you have not been  able to concentrate on things,  such as reading books,  newspapers, or watching TV in  the last 2 weeks? | No | A few days but not many | More than a week | Almost every day |
| Have you felt that your actions have  slowed down such as speaking  slowly/deliberately, so as to  attract the attention of others or,  on the contrary, you are fidgety,  irritable, and more likely to move  around than usual in the last 2  weeks? | No | A few days but not many | More than a week | Almost every day |
| Have you had any thoughts of  giving up on life, or hurting  yourself in the last 2 weeks? | No | A few days but not many | More than a week | Almost every day |
| If you have the above problems,  do they have any effect on your  usual work, family life, and how  you get along with people? | No impact | Some impact | Very impactful | Great impact |

Appendix 2. Baseline characteristics of groups in the follow-up visit.

| Variables | Completed the follow-up visit | |  | Deceased | |
| --- | --- | --- | --- | --- | --- |
|  | Mean | St. Dev. |  | Mean | St. Dev. |
| Key explanatory variable and control variables |  |  |  |  |  |
| Age (years) | 52.4962 | 8.6099 |  | 62.4602 | 8.1026 |
| Sex  (Men=1, Women=2) | 1.5488 | 0.4977 |  | 1.3356 | 0.4730 |
| Residence location  (Urban areas=1, 0 Otherwise) | 0.5311 | 0.4991 |  | 0.4637 | 0.4995 |
| Marital status  (Married=1, Divorced or living alone=0) | 0.8764 | 0.3292 |  | 0.8028 | 0.3986 |
| Employment status | 0.4636 | 0.5005 |  | 0.2561 | 0.4372 |
| (Employed=1, 0 Otherwise) |  |  |  |  |  |
| Cigarette smoking | 0.2352 | 0.4941 |  | 0.1869 | 0.3905 |
| (Current smoking=1, 0 Otherwise) |  |  |  |  |  |
| Baseline chronic diseases status |  |  |  |  |  |
| Type 2 diabetes  (Yes=1, 0 Otherwise) | 0.0600 | 0.2374 |  | 0.1384 | 0.3459 |
| Hyperlipidemia  (Yes=1, 0 Otherwise) | 0.0618 | 0.2410 |  | 0.0519 | 0.2222 |
| Hypertension  (Yes=1, 0 Otherwise) | 0.0571 | 0.2321 |  | 0.1107 | 0.3143 |
| Observations* | 5671 | |  | 289 | |

* Participants with incomplete physical examination or omission of questionnaire items in the baseline

survey were excluded.
